# AI and Data-Driven Advancements in Industry 4.0

**DOI:** 10.3390/s25072249

**Published:** 2025-04-02

**Authors:** Yan Pang, Teng Huang, Qiong Wang

**Affiliations:** 1 Shenzhen Institute of Advanced Technology, Chinese Academy of Sciences, Shenzhen 518055, China; wangqiong@siat.ac.cn; 2 School of Artificial Intelligence, Guangzhou University, Guangzhou 510700, China; huangteng1220@gzhu.edu.cn

## 1. AI in Industry

Industrial artificial intelligence is rapidly evolving, driven by an unprecedented explosion of diverse data modalities. In today’s smart factories and Industry 4.0 environments, vast streams of data—from machine logs and sensor readings to high-resolution imagery and acoustic signals—are fueling a new wave of data-centric innovation [1]. Organizations now collect heterogeneous datasets—ranging from natural language documents [2] and medical images [3] to sensor readings [4] and satellite imagery—to monitor, optimize, and transform industrial processes on an unprecedented scale.

This surge in data availability has sparked a fundamental shift toward a data-centric AI paradigm, where enhancing data quality and coverage becomes the primary driver of performance Zha et al. [5]. Unlike traditional model-centric approaches, data-centric strategies focus on meticulously curating multi-modal datasets, refining annotations, and ensuring that the data reflect the complexity of real-world industrial environments, thereby bolstering model learning.

Recent breakthroughs in deep learning are further accelerated by the rich, multi-sensor data now accessible in industrial settings—spanning high-resolution RGB images, LiDAR scans, hyperspectral imagery, and audio streams captured from various production sources Paheding et al. [6]. As a result, modern AI systems are beginning to leverage this wealth of multi-modal information to achieve superior generalization and even exhibit emergent capabilities once thought beyond reach. For example, large-scale multimodal models that integrate vision and language are now capable of generating detailed narratives about production processes or solving complex operational tasks without explicit instruction Baltrušaitis et al. [7]. In parallel, advancements in natural language processing (NLP), driven by scaling both models and data in large language models (LLMs), are unlocking novel behaviors, such as few-shot reasoning and chain-of-thought problem solving, which are increasingly relevant to industrial applications Xu et al. [8]. Contribution 1 introduces an innovative intelligent sensor software that combines robust machine learning with interactive heat map visualization to optimize resistance spot welding for steel reinforcement. Contribution 2 presents an artificial neural network (ANN) model that accurately predicts process performance. Contribution 3 introduces an innovative U-shaped network that integrates a Space-To-Depth module and a refined attention mechanism to effectively address class imbalance and achieve precise segmentation of micro and fine scratch defects on metal surfaces. These developments highlight how the diversity and volume of data are transforming AI techniques, establishing a robust foundation for the impactful deployment of industrial artificial intelligence across modern manufacturing and beyond.

The convergence of powerful AI methods with Industry 4.0 is driving transformative changes across a wide range of application domains Rai et al. [9]. Computer vision and NLP algorithms, in particular, are now deeply embedded in sectors such as healthcare Hirsch et al. [10], manufacturing Ahmad and Rahimi [11], and cybersecurity Zolanvari et al. [12]. In healthcare, deep learning models can analyze medical images (e.g., radiographs, MRI scans, pathology slides) with accuracy approaching or even exceeding expert human performance Shen et al. [13], thus aiding in diagnostics and treatment planning. Likewise, NLP techniques are used to mine clinical texts and biomedical literature Juhn and Liu [14], enabling predictive analytics and decision support in clinical workflows. These developments reflect a broader trend in which deep learning has fundamentally reshaped how we tackle tasks in vision, language, and healthcare analytics, among other fields (Paheding et al. [6], Abramson et al. [15]).

### 1.1. AI in Robotic

In recent years, the integration of artificial intelligence with robotics has led to transformative advancements in robotic perception, planning, decision making, and control Du and Yu [16], Sünderhauf et al. [17]. In medical applications, for instance, the fusion of imaging, localization, and magnetic actuation systems has enabled highly precise in vivo operations with miniature robots Aziz et al. [18]. In industrial and service contexts, visual and multimodal sensor fusion have significantly enhanced the robustness of grasping, navigation, and human–robot collaboration tasks Mao et al. [19]. Moreover, inspired by insights from neuroscience, brain-inspired robotics is exploring novel paradigms that emulate biological cognitive processes, thereby facilitating end-to-end autonomous learning and real-time adaptive control Qiao et al. [20]. These developments collectively represent a shift away from traditional, hand-coded control schemes toward intelligent systems that are capable of operating in complex, unstructured, and dynamic environments Hayes-Roth and Thorndyke [21]. Contribution 4 offers a comprehensive narrative review that elucidates machine learning strategies for the development of remotely monitored central nervous system biomarkers using wearable sensors. Contribution 5 presents an innovative task selection and allocation scheme for mobile crowdsensing that integrates Lyapunov optimization with a Double Deep Q-Network framework to dynamically stabilize task queues and optimize resource allocation under constrained conditions. Contribution 6 exemplifies the cutting-edge integration of multi-view imaging and advanced keypoint detection techniques to enhance robot arm monitoring in intelligent industrial environments.

### 1.2. AI in Medicine

The integration of artificial intelligence (AI) into healthcare within the framework of Industry 4.0 has revolutionized traditional medical practices, enabling data-driven decision making and personalized patient care. By leveraging advanced technologies such as machine learning and predictive analytics, AI facilitates the interpretation of complex medical data, including imaging diagnostics, where it enhances accuracy in identifying patterns in X-rays and MRI scans. This transformation aligns with the vision of Health 4.0, which emphasizes decentralized, AI-enabled home healthcare services, reducing reliance on conventional hospital-centric models. However, scaling AI applications in medical device manufacturing and clinical workflows remains challenging due to interoperability issues, regulatory hurdles, and the need for robust data governance frameworks. These challenges highlight the critical role of Industry 4.0 technologies in optimizing healthcare systems through digitization, automation, and real-time data exploitation.

The integration of artificial intelligence (AI) into healthcare within the framework of Industry 4.0 has revolutionized traditional medical practices, enabling data-driven decision making and personalized patient care Topol [22]. By leveraging advanced technologies, such as machine learning and predictive analytics, AI facilitates the interpretation of complex medical data, including imaging diagnostics, where it enhances accuracy in identifying patterns in X-rays and MRI scans Esteva et al. [23]. This transformation aligns with the vision of Health 4.0, which emphasizes decentralized, AI-enabled home healthcare services, reducing reliance on conventional hospital-centric models Keesara et al. [24]. However, scaling AI applications in medical device manufacturing and clinical workflows remains challenging due to interoperability issues, regulatory hurdles, and the need for robust data governance frameworks Rajkomar et al. [25]. Contribution 7 exemplifies a rigorous methodological advancement by integrating multi-scale feature extraction and channel selection to significantly enhance EEG-based Parkinson’s disease classification performance. Contribution 8 highlights the growing role of machine learning in oncology, with a focus on medical image analysis, treatment planning, and patient prognosis. Contribution 9 leverages a novel cross-scale attention mechanism alongside a progressive edge refinement module to enhance retinal vessel segmentation performance. These challenges highlight the critical role of Industry 4.0 technologies in optimizing healthcare systems through digitization, automation, and real-time data exploitation.

### 1.3. AI in Blockchain

In parallel, blockchain technology is intersecting with AI to enhance trust, security, and data integrity in Industry 4.0 applications Rahman et al. [26]. Blockchain (distributed ledger) systems provide tamper-resistant record keeping and decentralized data governance, which complement AI’s data-driven decision making. The amalgamation of AI and blockchain is recognized as a disruptive force of the Fourth Industrial Revolution, holding tremendous potential to create new business models and ecosystems Kumar et al. [27]. For instance, in industrial supply chains, blockchain can verify the provenance and authenticity of goods while AI monitors quality and demand forecasts Liu et al. [28]; in healthcare, blockchain enables the secure sharing of medical data or model parameters across institutions, while AI algorithms analyze those data for insights. Similarly, in finance and digital asset management, blockchain-based smart contracts can work in tandem with AI models to automate decision logic in a transparent, fraud-resistant manner Bathula et al. [29]. Early studies show integrated AI-blockchain platforms being explored for secure IoT networks, intellectual property protection for AI models and data, and federated learning setups where blockchain logs and verifies each update Issa et al. [30]. Contribution 10 combines Groth16 zero-knowledge proofs, blockchain technology, smart contracts, and IPFS to ensure secure. Contribution 11 introduces a novel deep learning and multimodal decision fusion approach that integrates source code, opcode, and control flow information to achieve high detection accuracy for smart contract vulnerabilities. This convergence of AI with decentralized technologies underscores the wide deployment of next-generation AI methods well beyond conventional settings, reaching into cyber-physical systems, smart cities, and global digital ecosystems that define Industry 4.0.

## 2. Trustworthy AI

As AI becomes deeply embedded in Industry 4.0 systems, ensuring that models are trustworthy, robust, and secure is of paramount importance. Real-world industrial AI applications (whether in autonomous driving, medical diagnosis, or power grid management) demand high reliability and resilience to adversities. However, recent research has revealed that AI models can be vulnerable to a variety of attacks and failure modes Hu et al. [31]. For example, adversarial attacks involve subtle perturbations to inputs that cause misclassification, data poisoning injects malicious data into training sets to subvert model behavior, and model inversion or extraction attacks can leak sensitive information from a model’s learned parameters. Such risks pose serious concerns in safety-critical environments. A malfunctioning vision system in a robot or an altered prediction in a clinical AI tool could have dire consequences. Therefore, the development of secure and robust AI models has become a critical research focus. Methods for adversarial defense, robust training (e.g., data augmentation, ensemble methods), and anomaly detection are being actively investigated to harden AI models against these threats. Equally crucial is safeguarding data privacy: Industry 4.0 AI often operates on sensitive personal or proprietary data. Therefore, techniques such as federated learning, differential privacy, and secure multi-party computation are being employed to protect data while still enabling collaborative learning.

In tandem with the robustness, trust, and transparency of AI systems, these systems are also receiving increasing attention. Stakeholders must be confident that AI-driven decisions or predictions can be understood and justified—especially in domains such as healthcare and manufacturing where accountability is essential Murdoch et al. [32]. Efforts in explainable AI (XAI) aim to highlight the reasoning behind a model’s output, whether through interpretable model architectures or post hoc explanation tools. Moreover, governance frameworks for Trustworthy AI have emerged Rudin [33], advocating principles such as fairness, accountability, transparency, and ethics in AI system design. For instance, regulators and industry consortia are beginning to recommend or require that AI systems provide audit trails of how data are used and how decisions are made Gunning et al. [34]. In manufacturing, this might mean that an AI quality inspection system can highlight which product features led to a rejection; in smart healthcare, an AI diagnostic assistant should explain its recommendation in terms a clinician can validate. Incorporating these principles mitigates the “black box” nature of many deep learning models and increases user confidence. In summary, achieving trusted AI in Industry 4.0 involves a multi-faceted approach: technical robustness against attacks and failures, privacy-preserving data practices, and transparency and fairness measures. Addressing these challenges is vital for the long-term sustainability and societal acceptance of AI-driven industrial systems.

## 3. Conclusions

In summary, this Editorial, titled “AI and Data-Driven Advancements in Industry 4.0”, has showcased a diverse array of studies that collectively underscore the pivotal role of AI and multi-modal data in modern industrial applications. Industrial automation, robotics, medical diagnostics, and blockchain integration are among the key areas explored, illustrating how advanced data-centric strategies and deep learning techniques are being harnessed to address complex challenges. These works exemplify how cutting-edge AI approaches not only enhance theoretical insights but also drive practical innovations in the evolving landscape of Industry 4.0.
